# Occupational Therapy Assessment for Upper Limb Rehabilitation: A Multisensor-Based Approach

**DOI:** 10.3389/fdgth.2021.784120

**Published:** 2021-12-17

**Authors:** Seedahmed S. Mahmoud, Zheng Cao, Jianming Fu, Xudong Gu, Qiang Fang

**Affiliations:** ^1^Department of Biomedical Engineering, College of Engineering, Shantou University, Shantou, China; ^2^The Second Hospital of Jiaxing, Jiaxing, China

**Keywords:** assessment, inertial measurement unit, kinect, occupational therapy, surface electromyograph, upper limb

## Abstract

Most post-stroke patients experience varying degrees of impairment in upper limb function and fine motor skills. Occupational therapy (OT) with other rehabilitation trainings is beneficial in improving the strength and dexterity of the impaired upper limb. An accurate upper limb assessment should be conducted before prescribing upper limb OT programs. In this paper, we present a novel multisensor method for the assessment of upper limb movements that uses kinematics and physiological sensors to capture the movement of the limbs and the surface electromyogram (sEMG). These sensors are Kinect, inertial measurement unit (IMU), Xsens, and sEMG. The key assessment features of the proposed model are as follows: (1) classification of OT exercises into four classes, (2) evaluation of the quality and completion of the OT exercises, and (3) evaluation of the relationship between upper limb mobility and muscle strength in patients. According to experimental results, the overall accuracy for OT-based motion classification is 82.2%. In addition, the fusing of Kinect and Xsens data reveals that muscle strength is highly correlated with the data with a correlation coefficient (CC) of 0.88. As a result of this research, occupational therapy specialists will be able to provide early support discharge, which could alleviate the problem of the great stress that the healthcare system is experiencing today.

## Introduction

Cerebrovascular accident also known as stroke has become the second most common cause of death and the leading cause of ongoing disabilities worldwide (1, 2). China has the largest stroke population in the world, with a total of over 13 million (3). In the last two decades, no country has experienced a decline in the number of stroke patients (4). Stroke patients have unmet needs in areas such as reintegration into society, health-related quality of life (QoL), maintenance of activities, and self-efficacy (5). As stroke patients experience a loss of self-care ability, it is difficult for them to maintain their daily activities.

It has become increasingly important to assist stroke patients to become more independent through post-stroke rehabilitation. Occupational therapy (OT) is one of the important treatment modalities for post-stroke rehabilitation. OT refers to the application of focused and selected occupational activities to prevent or recover the loss of various degrees due to physical, mental, and developmental dysfunction or disability (6). It aims at helping patients become independent by improving their limb strength.

In previous studies, it has been proved that patients' motor function and self-care abilities are improved after they undergo OT. In (7), researchers proved that OT could significantly improve the ability of daily life of stroke patients. In (8), Qian et al. found that OT significantly improved stroke patients' motor function and also improved their daily activities and reduced their dependency. Researchers in (9) demonstrated that OT plays an important role in the intervention of stroke patients and also helping them to maximize their QOL. Studies in (10) found that OT was effective for stroke patients. Thus, OT has been shown to have good therapeutic effect, improve limb function and self-efficacy, and enhance QOL in patients; it is also believed to have good clinical promotion value.

Richards et al. in their study pointed out that OT has positive impact on the health and QOL of people with disabilities; however, the demand for OT services globally is difficult to fulfill (11). OT currently requires one-to-one guidance from rehabilitation specialists (12). The OT and upper limb assessment are resource-intensive processes that require the presence of an OT specialist. In this regard, automation of this process would result in a paradigm shift in the rehabilitation process. One of the most important problems of empirical assessment is that it lacks objective standards. In this paper, a multisensor-based upper limb assessment model is proposed. The measurement data captured by three different types of sensors are processed to assess the quality of the OT exercise or movement (called OT action onward) completed by a patient and to ensure that the patient performs the rehabilitation training session properly. Based on experimental results, the overall accuracy of the OT-based motion classification is 82.2%. Furthermore, combination of Kinect and Xsens kinematic data yields a correlation coefficient (CC) of 0.88 with muscle strength. This finding indicates that the degree of flexibility in the upper limbs is highly correlated with muscle strength. In addition, the proposed model shows promise in helping rehabilitation specialists assess upper limb function.

## Related Work

Researchers have widely used wearable sensors for upper and lower limb assessments (13–22). Table 1 presents a summarized overview of upper limb mobility assessment for post-stroke patients. In (13), the data captured by sensors were used to determine joint flexibility and limb mobility (13). We used a portable infrared imaging device to accurately measure multiple joint angles of a hand simultaneously in (13). In (14), researchers proposed an automated upper extremity motor function assessment system using Kinect v2 and force-sensing resistor sensors. Using the extracted features from the sensor data and the linguistic guidelines of the Fugl-Meyer Assessment (FMA), a rule-based binary logic classification algorithm was developed to assign FMA scores to upper limb motion data. FMA is one of the clinical upper extremities assessment methods that can be used to assess limb function. In their classification approach, a simple decision classification method was used to evaluate each FMA test using 3-point scale (0: cannot be performed at all; 1: can be performed partially; and 2: can be performed faultlessly). Clinical trials with nine stroke patients produced an accuracy rate of 90%. The accuracy rates of some FMA tests were as low as 66.7% due to insufficient patient data.

**Table 1:** Overview of upper limb movements assessment for post-stroke patients.

**Reference**	**Sensors fusion** **(Measurement error reduction)**	**Assessment method**	**Sensor type**	**Description**
Qiang et al. (13)	None	Swanson, Fugl-Meyer	Leap motion (non-touch)	Hand function assessment. Measuring ROM for hand and also multiple joint angles of a hand.
Lee et al. (14)	None	Fugl-Meyer	Kinect v2, force-sensing resistor (non-touch)	Upper limb ROM assessment using fused classifiers and FMA. Muscle strength was not examined.
Zhang et al. (15), Zhang et al. (16)	None	Brunnstrom, MI	IMUs (wearable)	Upper limb assessment using hybrid algorithm combining PCA and fuzzy inference system.
Qiang et al. (17)	None	Brunnstrom, MI	IMUs (wearable)	60 weeks of longitudinal investigation of in-home upper limb assessment. Rehabilitation recovery trend demonstration.
Akbari et al. (18)	Fused	Limb angles measurement	IMUs, Kinect (wearable and non-touch)	Measurement error was reduced by fusing IMU and Kinect sensors' data. Muscle strength was not examined.
Wang et al. (19)	None	Quantitative assessment	Kinematic data, sEMG (wearable)	Two types of single-modality classifiers (i.e., local classifiers and ensemble classifiers) were created to evaluate upper limb motor function.
Liparulo et al. (20)	None	Brunnstrom	sEMG (wearable)	Fuzzy logic approach was used to evaluate stroke patients' impairment level.

An in-home upper limb rehabilitation system using two IMU sensors was proposed in (15, 16). The two IMU sensors were used for upper extremities kinematic analysis. A mobility index (MI) algorithm which was found to be highly correlated with Brunnstrom classification was considered as a major feature of the in-home rehabilitation system (15, 16). A hybrid algorithm combining principal component analysis (PCA) and fuzzy inference system was used for the upper limb impairment level evaluation. The deployment and longitudinal trial of the supported in-home post-stroke rehabilitation in (15, 16) is presented in (17). The longitudinal investigation lasted for 60 weeks and involved 12 patients within their first 3 months from stroke event. The results demonstrated that the patients from the experimental group had experienced a steady increase in MI throughout the program.

Research in (18) combined data from IMUs and Kinect sensors to measure upper and lower body motions. An extended Kalman filter (KF) was used during the fusion process of the two sensors. It was found that by combining two sensors' data, Kinect- and IMU-based sensors, the assessment error could be reduced; therefore, the assessment accuracy improved (18). Several researchers have used surface electromyography (sEMG) data to analyze upper limb motor function. In (19), Wang et al. used Kinematic and sEMG data for the assessment of upper limb of post-stroke hemiparetic patients. Kinematic data and sEMG signals were collected simultaneously from participants, and motor features extracted from each sensor were fed into local classifiers. Two types of single-modality classifiers (i.e., local classifiers and ensemble classifiers) were created to evaluate upper limb motor function (19). The multimodal fusion classification scheme exhibited an accuracy of 96 % in distinguishing pathological movements from normal movements. In (20), sEMG was used to classify and evaluate patients' upper limbs using Brunnstrom stages evaluation techniques. Moreover, researchers in (21) used an sEMG and an IMU data to study the quality of the OT actions during OT. Their research differentiates between the movement of affected limb and the movement of healthy limb. These models were aimed to assist in the assessments of movements during OT.

In previous research, the classification of OT actions overlooked the quality and completeness of the exercises. In addition, previous research did not investigate and correlate muscle strength with upper limb motion. An upper limb evaluation method that is capable of both assessment tasks is required. The findings of multisensor assessment methods motivate the investigation in this paper where a Kinect, IMU, and sEMG are used to assess upper extremities. In this research, we choose patients' kinematic data and also physiological data to assess upper limb motion during OT sessions. Kinect and Xsens are used to obtain the kinematic data whereas Biopac Multichannel Physiometer are used to capture an sEMG data. In this paper, Kinect and Xsens/IMU data are combined using a correlation measurement technique to determine the completion of specific OT action whereas sEMG is used for the classification of the OT actions. Using the three sensors' data, the proposed model can classify the OT action types and also to determine the completion of the actions during OT sessions.

## The Proposed Multisensor Assessment Method

**Figure 1:**
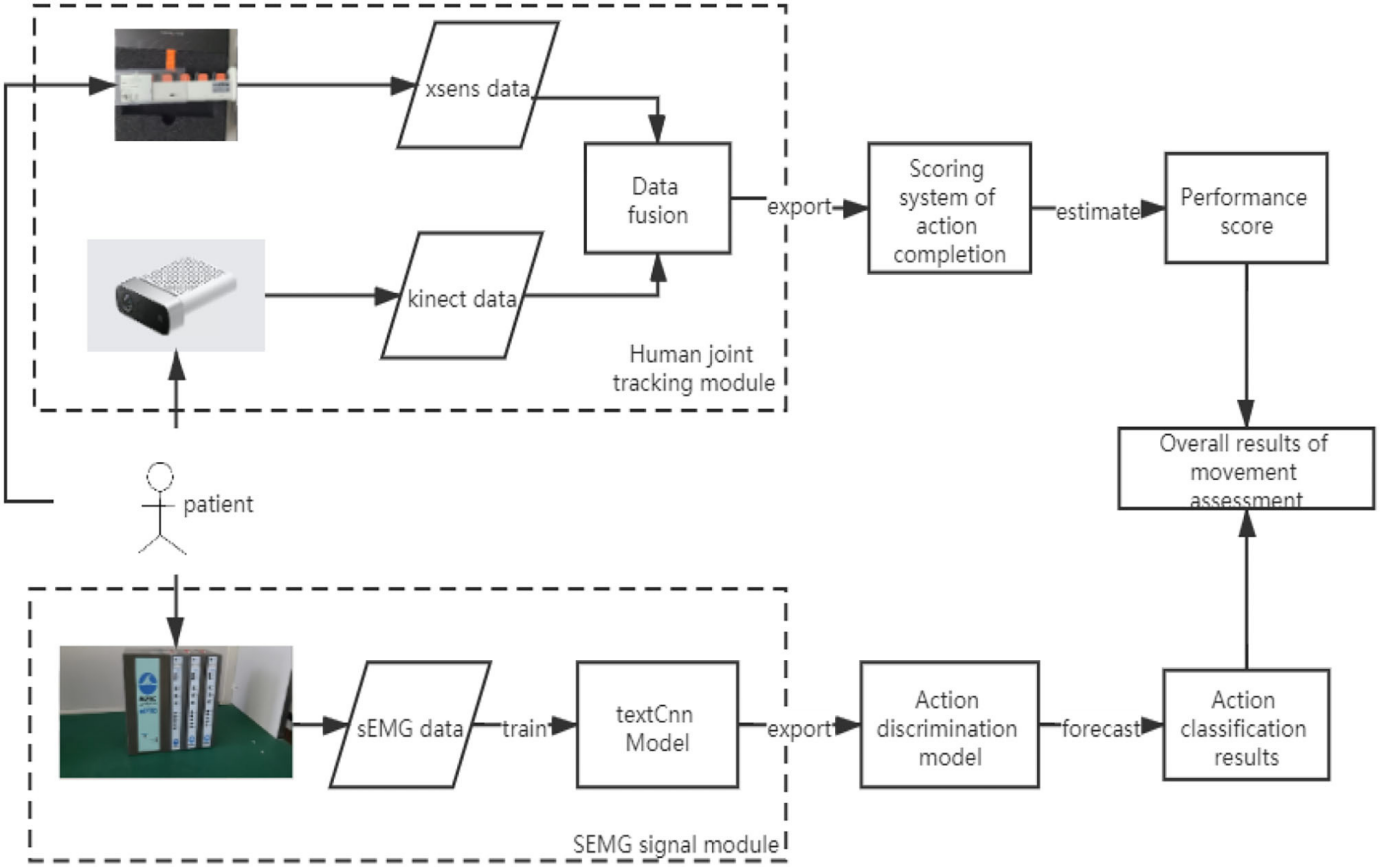
The schematic diagram of the proposed upper extremities assessment model.

A multisensor assessment method for upper limb will be presented in this section. The proposed method used kinematic information and physiological data to classify the OT rehabilitation actions and also to determine the completion and quality of the actions during OT sessions. Figure 1 shows the schematic diagram of the proposed upper limb assessment model based on OT actions. In this model, there are two main modules. These modules are the human joint tracking module and the sEMG signal module. Kinematic data captured by Kinect and Xsens are fused and correlated to measure the completion level of specific OT actions. The classification of OT action is achieved by the classification of the sEMG signal using convolutional neural network (CNN) with a modified TextCNN model.

### Kinect and Xsens Fusion Algorithm

Azure Kinect is a depth camera sensor launched by Microsoft™. It provides human tracking software development kit (SDK), which can conveniently provide the spatial coordinates of human joints.

**Figure 2:**
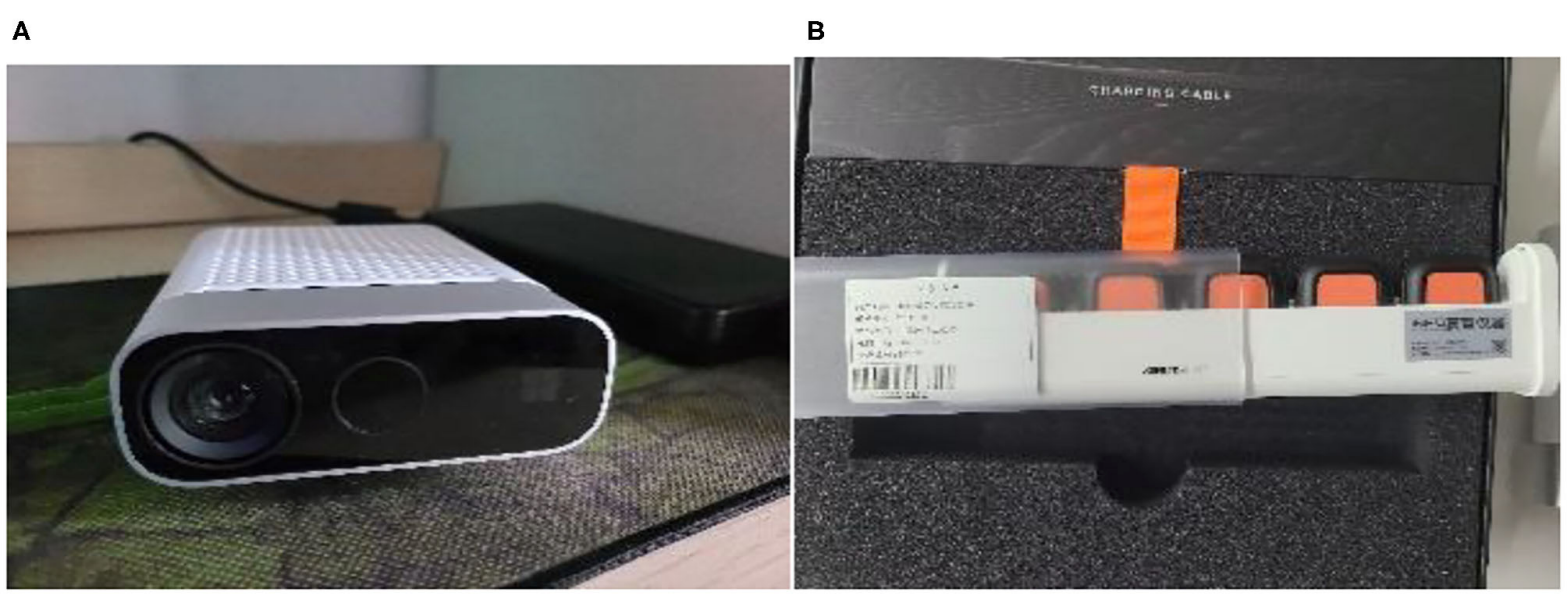
The motion capture sensors. **(A)** Azure Kinect, **(B)** Xsens Dot.

The IMU sensor uses the Xsens Dot package developed by Xsens, which can provide acceleration and angular information with sampling frequency at 800 Hz. A precise time alignment between Kinect and Xsens data during OT exercises is crucial. Data from Kinect and Xsens include timestamps for each sample. The synchronization is performed by aligning the two sensors' timestamps. Data from both sensors are examined, synchronized, and fine-tuned manually. To prevent data loss during synchronization, extra data were collected before and after the OT exercises. The fusion of the two synchronized sensors can reduce the measurement error caused by the two sensors, thus improving the quality of kinematic data. Figure 2 shows the two motion sensors used in this study. Mostly, researchers use KF to merge or fuse sensor data to reduce measurement error (18, 22). This paper uses KF to fuse the upper limb data captured by both the Kinect and the Xsens sensors.

#### Coordinates Information for Kinect and Xsens

**Figure 3:**
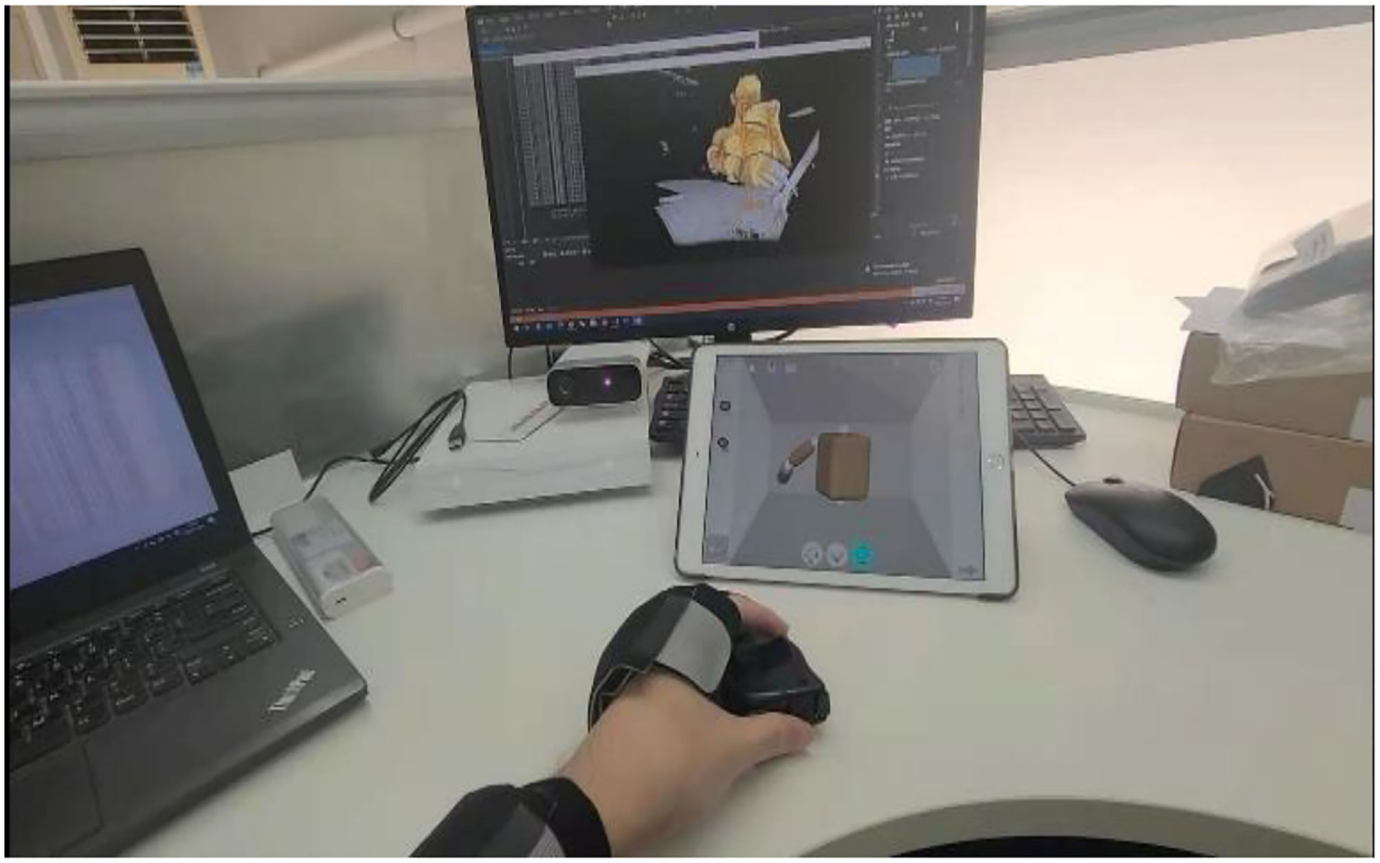
Kinect and Xsens data collection setup.

Generally, sensor measurement inherits errors. To reduce the error, the idea of fusing two sensors' data is proposed. Xsens Dot uses related derivative software, Kinexyz, to provide the spatial coordinate data whereas Kinect directly calls the joint coordinate data using the method and data collection process shown in Figure 3.

**Figure 4:**
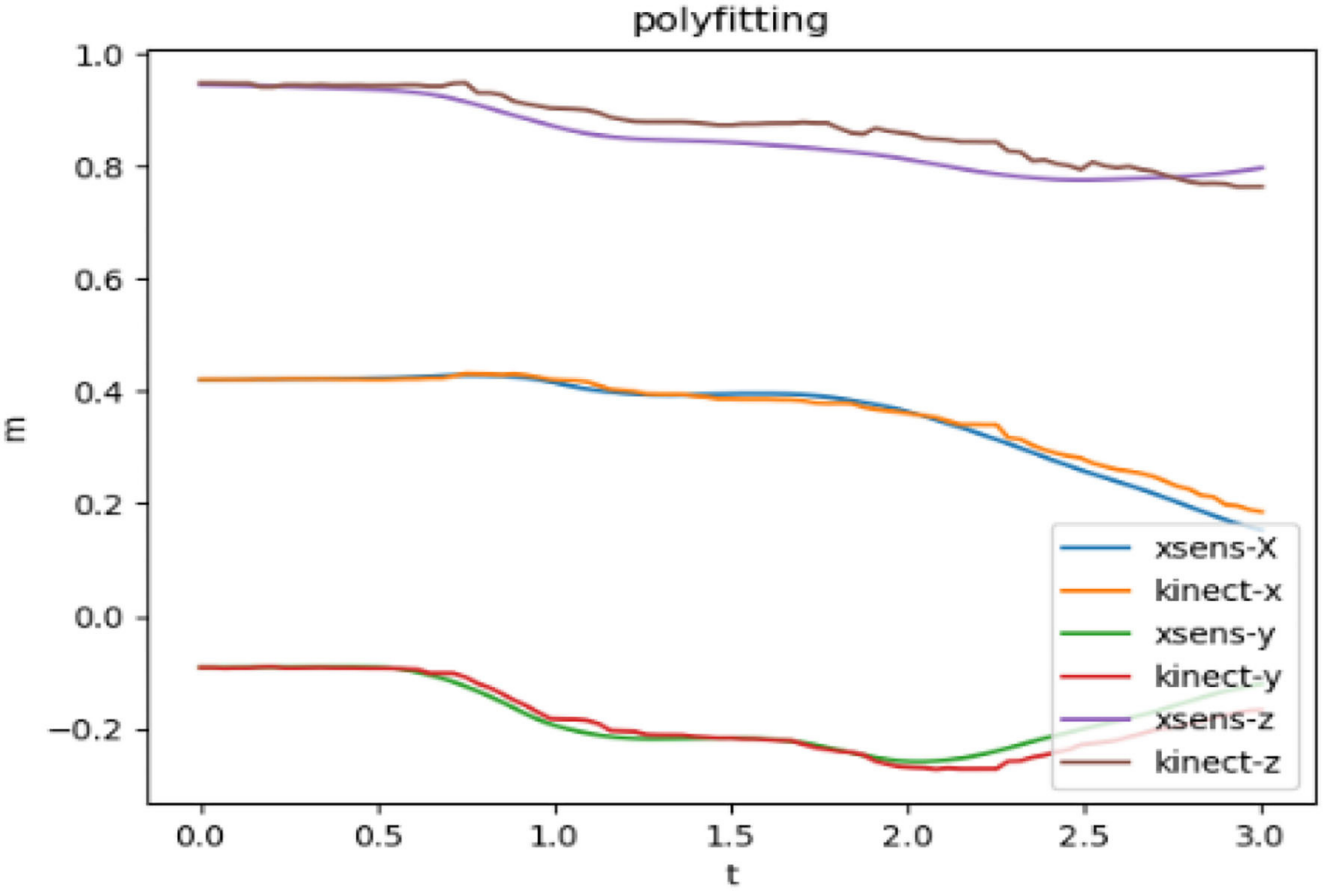
Kinect and Xsens coordinate data.

Figure 4 shows the limb's joint data that are collected by Kinect and Xsens sensors independently, before the data fusion. The t-axis represents the time, the m-axis represents the coordinate value, and the xyz are the values in the three directions of the space coordinate axis.

#### Kinematic Data Fusion by KF

**Figure 5:**
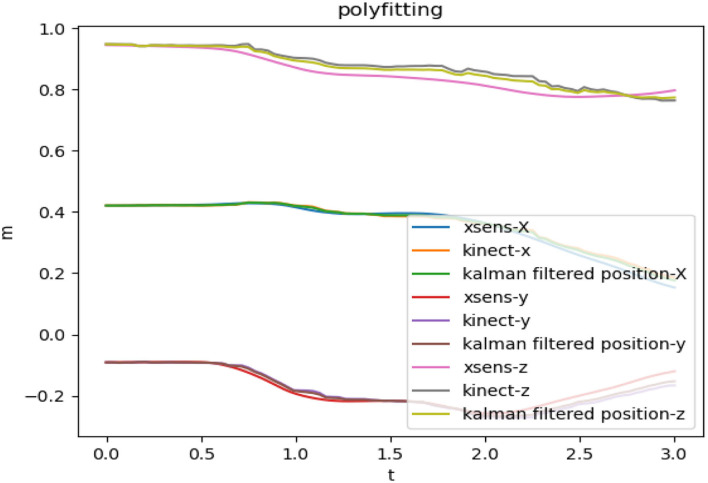
Upper limb joint movement data after using KF.

Kalman filter, is an algorithm, uses for optimal system's parameter estimation (23). It achieves the optimal estimation by predicting value from current value and previous observed value. Calculation of Kalman gain is required to achieve optimal parameter estimation. The KF is iterated continuously over time, and the optimal estimate is finally obtained by updating the Kalman gain. The KF algorithm used in the two sensors' fusion process is given by the pseudocode in Algorithm 1. Figure 5 shows the upper limb motion data for the two sensors, and also the fused data using KF.

**Algorithm 1:** **Kinematic data fused with Kalman filter**.

*// KF uses Kinect and Xsens data for patients' movement*
*// optimal estimation. For upper limb motion*
**1: Start**
**2: Capture:** Kinect measurement *a*, Xsens measurement *b*
**3: Measure the variance** *A* of Kinect data
**4: Initialization →** prediction data *p*_0_, noise *Z* (variance of Xsens)
**5: Calculate Xsens Error:** P_k_' = (p0+Z) ^1/2^
**6: Calculate Kalman gain:** K_k_= (P_k_'^2^ / (P_k_'^2^+A)) ^1/2^
**7: Produce predicted value:** x = a + K_k_ * (a-b)
**8: Variance of update forecast:** P_K_= ((1-K_k_) *P_k_' ^2^) ^1/2^
**9: Iterate above steps to update Kalman gain**
**10: Optimal estimation is achieved**
**11: End**

### Upper Limb Movement Completion Analysis During OT

Patients' joint spatial movement data are acquired by the kinematic sensors including the Kinect and the Xsens for upper limb motion completion assessment analysis. The motion data are segmented into different motion group types for each patient and subject. The analysis in this method is based on the correlation between healthy subjects' standard motion and patients' motion for each OT action type. The CC represents the motion completion level for specific motion type performed by the patient. Dynamic time wrapping (DTW) (24) is used with the CC for the upper limb movement completion. Also, DTW is used in the motion completion algorithm to mitigate the situation when an uneven segmentation is presented in the preprocessing stage. An uneven segmentation may result in high similarity and low CC. In this paper, Pearson CC, r, is used and given by


(1) $$\begin{array}{r} r\left(x1,x2\right)=\frac{cov\left(x1,x2\right)}{\sqrt{var\left(x1\right)var\left(x2\right)}} \end{array}$$

where the variable *x*1 is the healthy subject's fused kinematic movement data and *x*2 is the patient's fused kinematic movement data.

The OT movement completion assessment is based on the significance of the Pearson CC. The scale of the CC is divided into multiple ranges to represent the correlation between the patient's spatial data and the healthy subject's spatial data. These ranges are used to score the completion of the limb's joint movement. The ranges and the corresponding scores are shown in Table 2. An absolute value of the CC below 0.3 represents insignificant correlation (scored as incomplete action, 0 score), CC in the range of 0.3–0.5 represents low correlation (poor action, 1 score), CC of 0.5–0.8 represents significant correlation (moderate correlation, 2 score), and CC of 0.8 and above represents high correlation (excellent correlation, 3 score).

**Table 2:** Relationship between CC and action completion score.

**Correlation coefficient**	**OT action completion score**
*r* ≥ 0.8	3
0.5 ≤ *r* < 0.8	2
0.3 ≤ *r* < 0.5	1
*r* < 0.3	0

### Upper Limb OT Movements Classification

Surface electromyogram (sEMG) is used for the classification of the OT actions. The classification process includes automatic sEMG segmentation, filtration, and classification. Automatic sEMG segmentation and classification will be presented in the next sections.

#### Automatic Segmentation and Filtration of the sEMG

**Figure 6:**
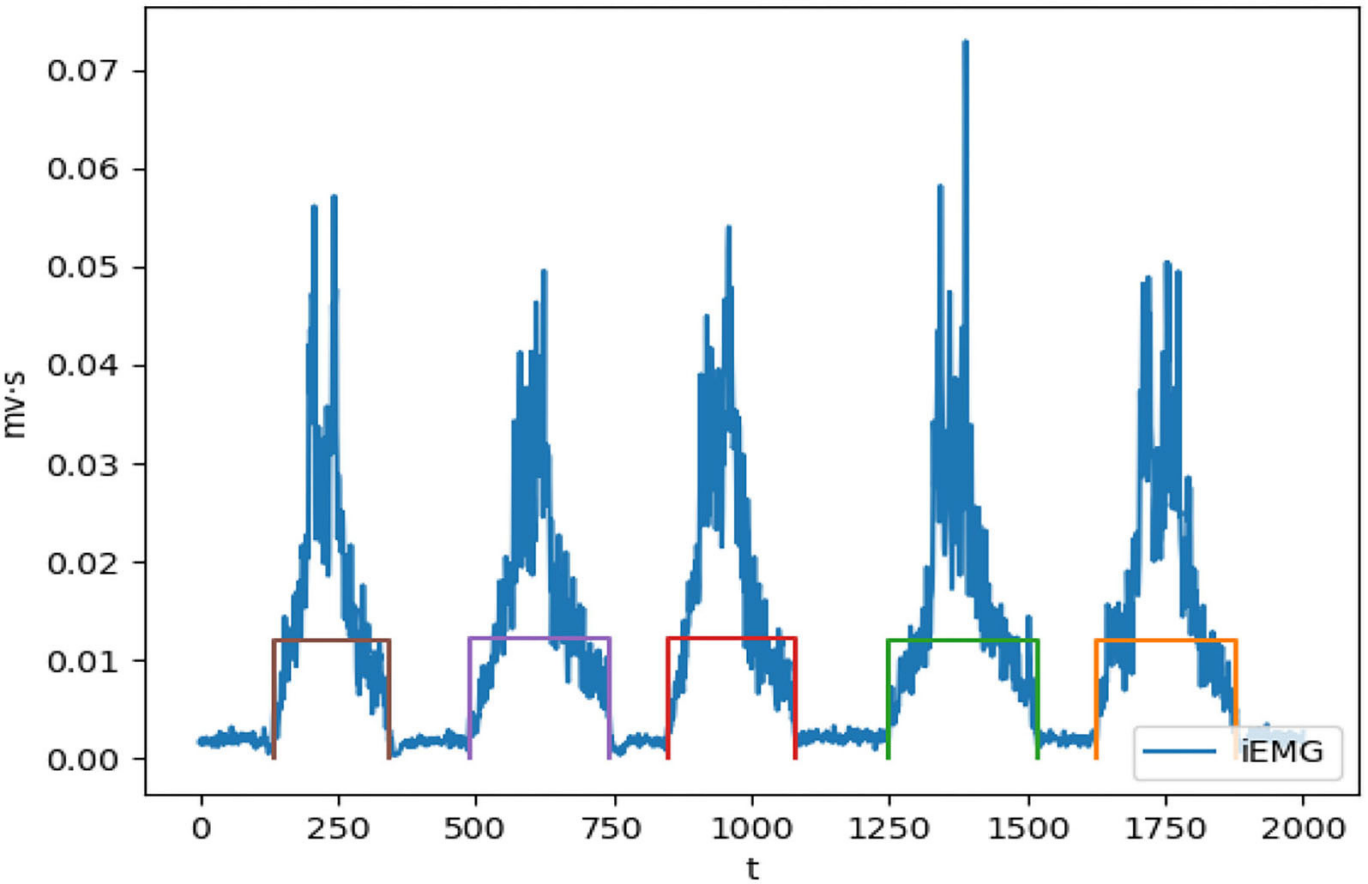
Conversion of sEMG to iEMG for motion segmentation.

For the classification of upper limb actions during OT session, an automatic segmentation of the sEMG event corresponds to limb motion during OT session, and filtration is required. The sEMG is acquired *via* the Biopac MP150. The sEMG's electrodes were placed at the upper forelimb muscle group, at biceps brachii region and at the deltoid region. The preprocessed sEMG is used as input to the classification model, TextCNN model (see Figure 1). The automatic segmentation algorithm is achieved by the integration of the original sEMG signal, the myoelectric integral (iEMG), to decide the start and end of the sEMG that correspond to specific OT action. The iEMG is defined as a time-windowed mean of the absolute value of the signal. Figure 6 shows the sEMG segmentation using the iEMG.

The algorithm for the automatic detection of the sEMG that corresponds to the movement activity is given by the pseudocode in Algorithm 2. The detected sEMG segment is filtered to remove unwanted noise.

**Algorithm 2:** **Automatic segmentation of upper limb motion**.

*// Myoelectric integral is used to automatically detect the sEMG*
*// Segmented sEMG should correspond to the motion of the upper limb*
**1: Start**
**2: Traverse the current sEMG array:** find the minimum value *a*, the time ID for the minimum, and the array length **L**
**3: Let:** max1 = a * L
**4: if** ID ≠ 0
**then**
intercept array [0, ID] to return step:1 for recursive operation
max2 = recursive return value.
**else**
max2=0.
**5: if** ID ≠ *L*−1
**then**
intercept array [ID, L] to return step:1 for recursive operation
max3 = recursive return value.
**else**
max3=0.
**6: Store maximums:** [max1, max2, max3]
**7: Returns maximums (**max1, max2, max3) **and their corresponding intervals**, ID.
**11: End**

#### Upper Limb Movement Classification Using TextCNN

**Figure 7:**
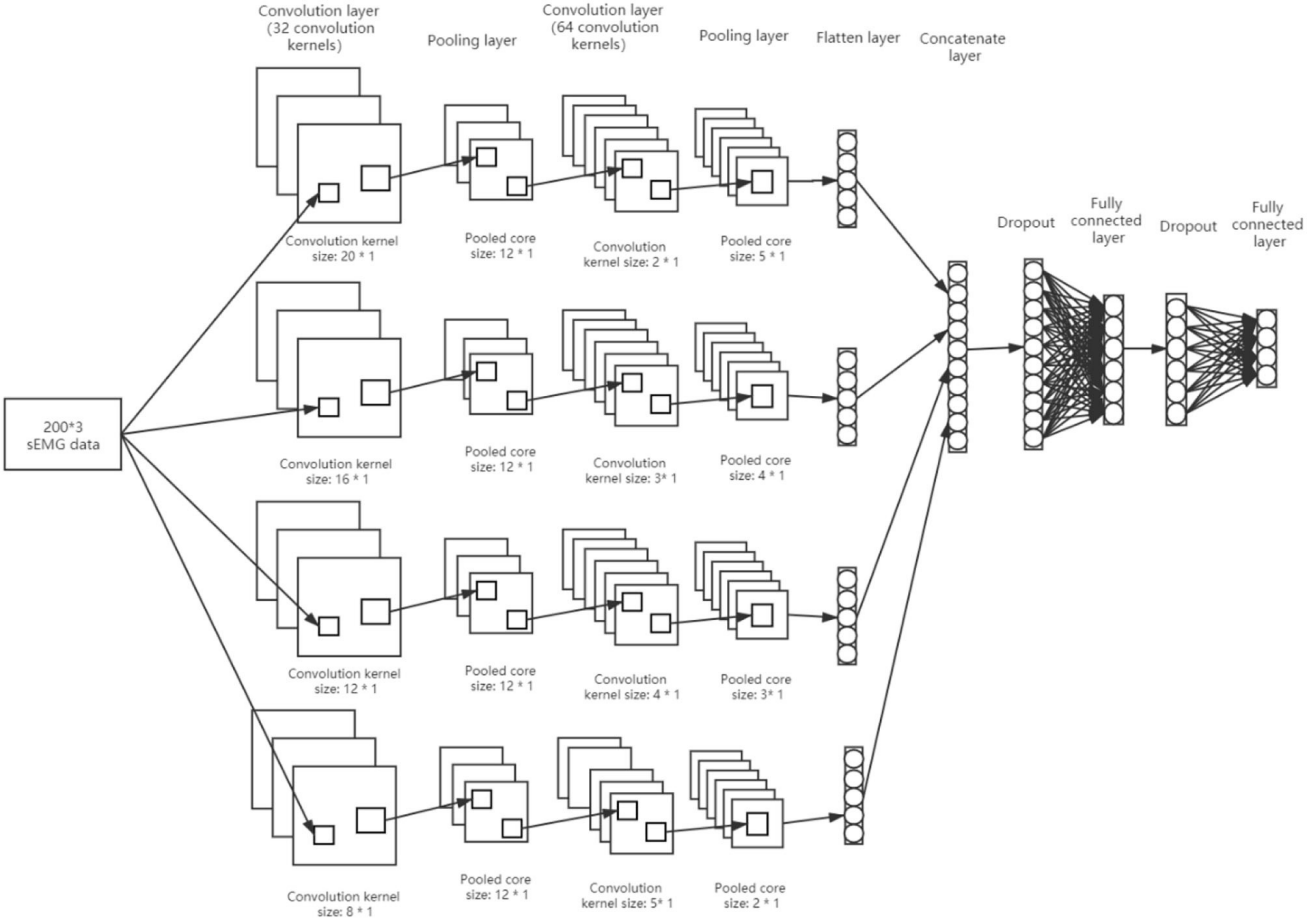
The modified version of the TextCNN classification model.

In (25), Kim proposed the TextCNN classification model. This model was used for text classification. It uses convolution kernels of different sizes to convolve the text input data. The kernel of different sizes can capture the correlation characteristics of different numbers of adjacent words. There is some similarity between the sEMG data and the text data when apply to this model. In this paper, the TextCNN model is expanded and modified as shown in Figure 7.

At first, the TextCNN is modified by adjusting the type of convolution core to four, and then the number of convolution pooling operation is adjusted to two. Moreover, an additional full connection layer is added to imitate the AlexNet model, and the dropout operation is used for the full connection layer. Also, the convolution core size of the first convolution operation is increased. The full connection activation at the output is four. By applying the sEMG data to the TextCNN model in Figure 7, the data are reduced during the testing and training of TextCNN. The size of the input sEMG data is 200 x 3 (there were three channels, each with 200 samples of sEMG data, for a total of 536 datasets). Initially, the data pass through a first convolution layer that has 32 convolution kernels with sizes of (20*1, 16*1, 12*1, 8*1). Through this process, the data were transformed into 32 feature maps of (181*3, 185*3, 189*3, 193*3). The output data from the previous layer are then passed through the first pooling layer, which has a pooling window size of 12*1. This layer produced 32 feature maps of (15*3, 15*3, 15*3, 16*3). Afterward, the output of the first pooling layer is fed into the second convolution layer, which consists of 64 convolution kernels of size (5*1, 4*1, 3*1, 2*1). In this layer, 64 feature maps were produced (14*3, 13*3, 12*3, 12*3). Data from the previous layer are then passed through the second pooling layer, which has pooling window sizes of (5*1, 4*1, 3*1, 2*1). The layer produced 64 feature maps of (2*3, 2*3, 2*3, 3*3). In the final step, the feature maps obtained from the second pooling are paved to yield (384, 384, 384, 576) eigenvalues, resulting in a total of 1,728 eigenvalues. Thereafter, four output nodes are created for the classification of the OT actions. By inserting dropouts to randomly hide some eigenvalues, the training process is completed.

Feature selection is critical to the success of any classification task. In this model, the features are selected from the sEMG through convolution processes. A selection of robust features influenced the TextCNN weights during training.

## Experimental Setup

Using post-stroke patient data, clinical experiments have been conducted to examine the performance of the proposed multisensor-based upper limb movement assessment system. The detail of the dataset and the experimental setup and protocol will be presented in this section.

### Participant

**Figure 8:**
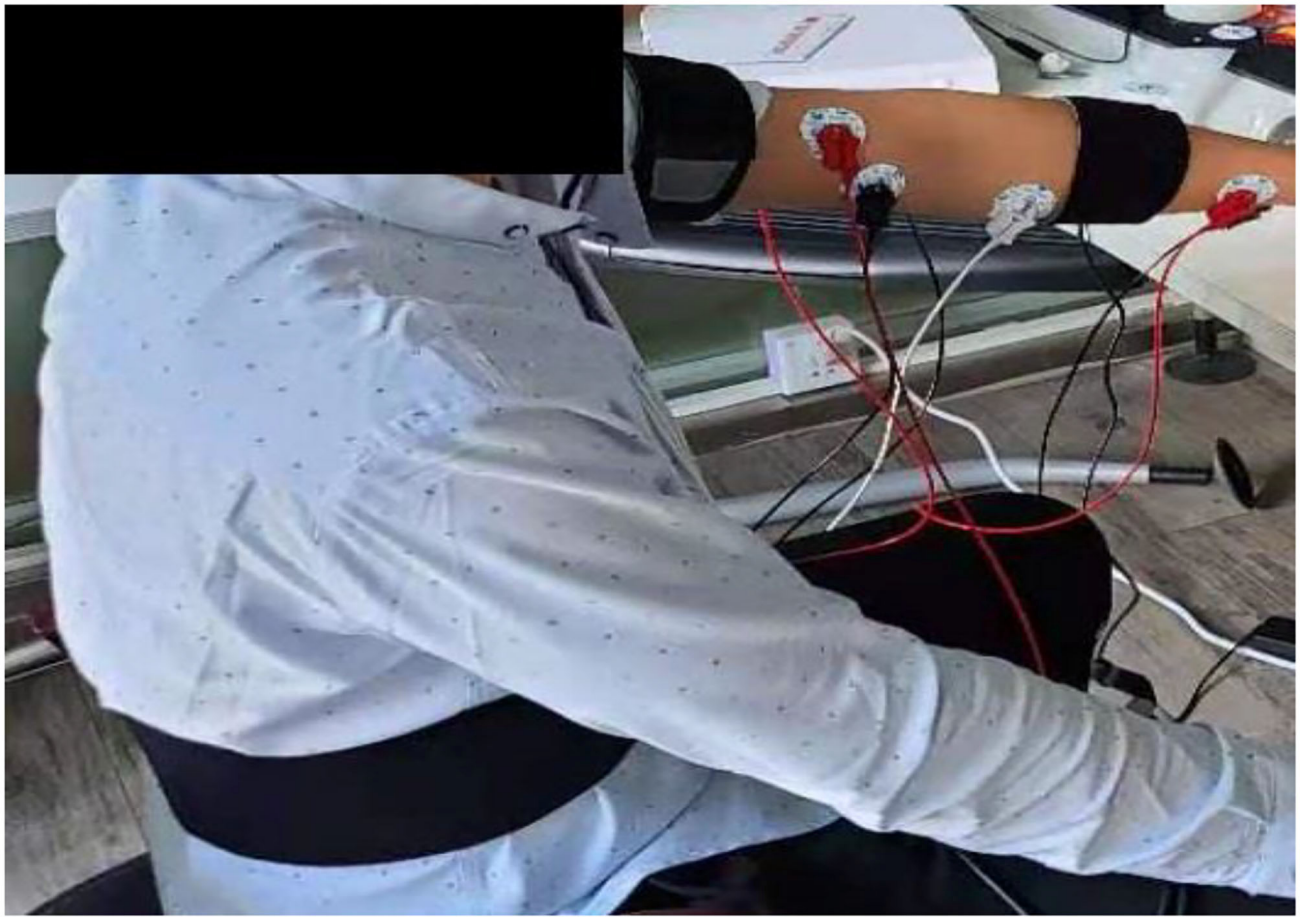
Xsens wearing position.

**Figure 9:**
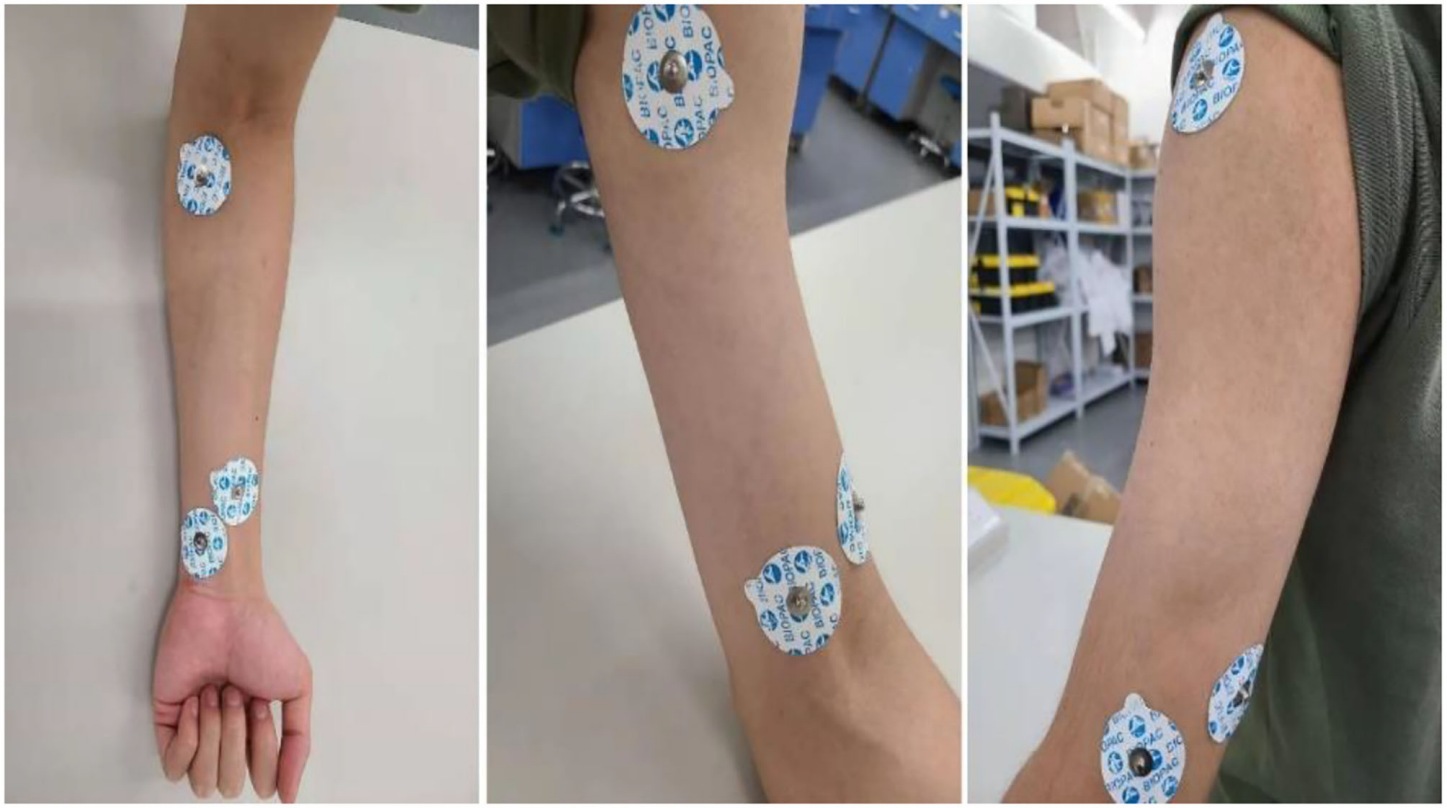
Electrodes placing position of three channels sEMG.

Nine healthy subjects (mean age: 21.5 ± 3.1 years) and nine patients (mean age: 58.2 ± 4.8 years) were recruited in this study. Healthy participants were part of this investigation to help in setting an upper limb mobility features benchmark. Particularly, their sEMGs were used to determine the reliability of the modified TextCNN model, whereas their motion data were used to determine the completion and quality of the actions during OT sessions as part of the post-stroke patient assessment. The patients were recruited from the Second Hospital of Jiaxing, Zhejiang province, China. The data collection was approved by the ethics committees of the hospital, and the procedures were strictly followed to ensure the investigation complied with the Declaration of Helsinki. The patients were verified to be within the phase of stroke recovery using computer tomography (CT) or magnetic resonance imaging (MRI). The criteria for including the patients were as follows: (1) no hemodynamic instability; (2) no severe cognitive impairments; (3) no dementia; (4) no major post-stroke complication; and (5) able and willing to give consent. These criteria are necessary to minimize the effects of a slight age difference within the patients' group. The age difference among the recruited patients is small in comparison with other studies in the literature (14). For example, in (14), the recruited patients have a mean age of 58.2 ± 4.8 years. In this study, sEMG and kinematic data were collected from patients and subjects. For kinematic data, a wearable sensor, Xsens, and non-wearable sensor, Kinect, are used. The Xsens is worn on the forelimb and hind limb of the hand and on the waist as shown in Figure 8. For sEMG, three channels are used. The sEMG's electrodes are attached to the muscle group of the forelimb, biceps region, and deltoid region of the hand as shown in Figure 9.

### Protocol

Four upper limb OT movement sets were performed by patients and healthy subjects. These OT movement actions aim to imitate various OT actions. The first set of action involves grabbing an object whose weight is fixed during all experiments. To perform this action, the patient must first stretch his/her arm while holding the object, then raise it over his/ her head at an angle of more than 45 degrees and slowly put it down. The second set of actions, pick up the object tremblingly, raise it to a certain height and lower it a little, then raise to a certain height and lower it again, and then raise it to the horizontal plane with an angle of >45 degrees and put it down. The third set of action is to grab the object straight with the arm lifted to the same horizontal plane for an angle of >20 degrees and then put it down, while the fourth set of action is to lift the object to a certain height, then move it horizontally to the right and then put it down. The four sets of OT actions are intended to simulate some daily activities to test a patient's motor skills. Characteristics of each group of OT actions are shown in Table 3.

**Table 3:** The characteristics of the designed OT movements or action.

**Action ID**	**Movement characteristics**
Action 1	To simulate the daily activity of moving objects to a high level and investigate the ability of the patient's arm with load to resist gravity.
Action 2	To simulate the action of cleaning the window, the stability of the patient's loaded movement was mainly investigated.
Action 3	To control action 1, the actual main investigation of the patient's arm in the state of low-load movement.
Action 4	To simulate the situation of daily horizontal movement of objects, mainly to investigate the ability of patients with horizontal arm movement.

The experiment protocol requested patients and subjects to perform at least 15 movements on each set of four OT actions; the overall number of actions per formed for the four groups is 60 times (15 × 4). Patients requested to rest for 2 s between each movement within the same OT action, then rest for 5 min after completing the 15 actions within the same OT action group. A total of 536 movements were recorded from nine patients, whereas 586 were recorded from healthy subjects.

For evaluating the correlation between patients' muscle strength and their fused motion data, three senior rehabilitation specialists each with more than 20 years of clinical practice evaluated and graded the nine patients' muscle strength scores. Lovett muscle strength grading standard was used (26). In this standard, grade 0 indicates that there is no movement observed whereas grade 5 indicates that the person's muscle contract normal against full resistance (healthy individuals) (26). Table 4 shows the muscle strength grade of each recruited patient.

**Table 4:** Muscle strength grade of each patient.

**Muscle strength grade**	**Patient number**
Grade 3	1, 2, 3, 8
Grade 4	5, 6, 7, 9
Grade 5	4

## Experimental Results and Discussions

In this paper, we proposed a multisensor-based upper limb movement assessment method. The main assessment features of the proposed method are as follows:

- A classification of patient's OT-based actions into four classes. Patients' sEMG data from the upper extremity were used in the OT-based multiclassification task.
- A measurement of quality of the patient's OT-based actions, OT action completion score. The assessment was conducted using Kinect and Xsens kinematic sensors.
- Evaluation of the relationship between upper limb mobility and muscle strength in patients.

In the proposed kinematic fusion technique, the Kinect and Xsens data were fused to obtain the most accurate estimation of the upper limb's joint movements. The fused motion data are then segmented and grouped in four OT actions and scored for action completion. The automatically segmented and filtered sEMG is used to classify and label relevant OT actions. The results for the completion of OT actions and its classification are shown in the next subsection.

### Upper Limb Movement Classification Performance Results

**Figure 10:**
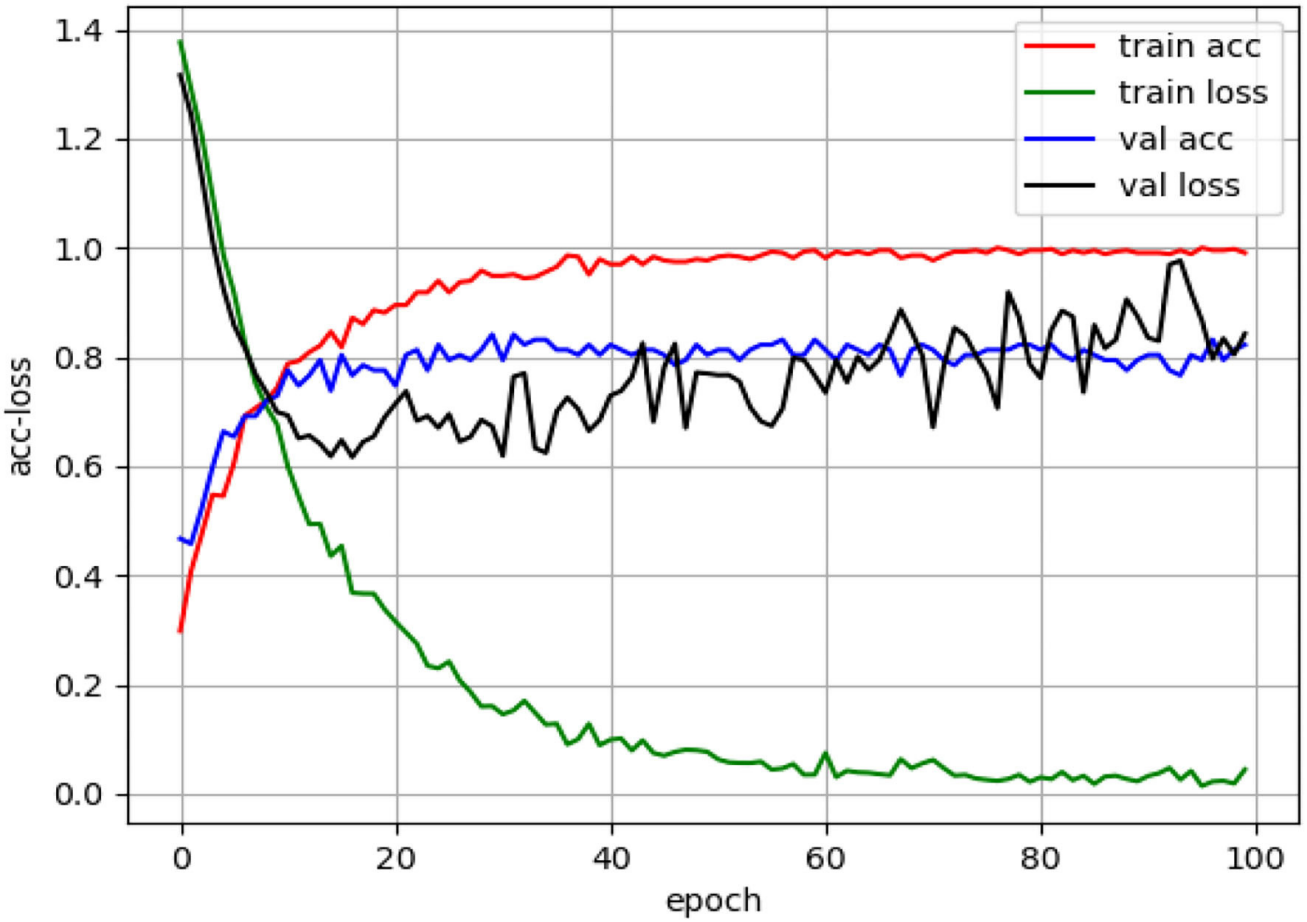
Modified-improved classification model performance during the training process with patients' dataset.

A sEMG signal is used as input to the classification model, modified-improved TextCNN. The parameters of the modified-improved TextCNN were set to produce an optimum classification performance. For patient's data classification, the sEMG is preprocessed to obtain a 536*200*3 dataset for three sEMG channels. In the movement classification model, sEMG of patients was used during training and testing. A train-test split method was used to estimate the TextCNN's performance. In this study, 80% of the data were used for training and 20% for testing. The data for the tests were randomly selected. Due to the small number of patients, each patient repeated OT exercises multiple times. The size of the data in each OT action increases using this method, thus improving classification model performance and reliability. Figure 10 shows the training loss and accuracy results.

**Figure 11:**
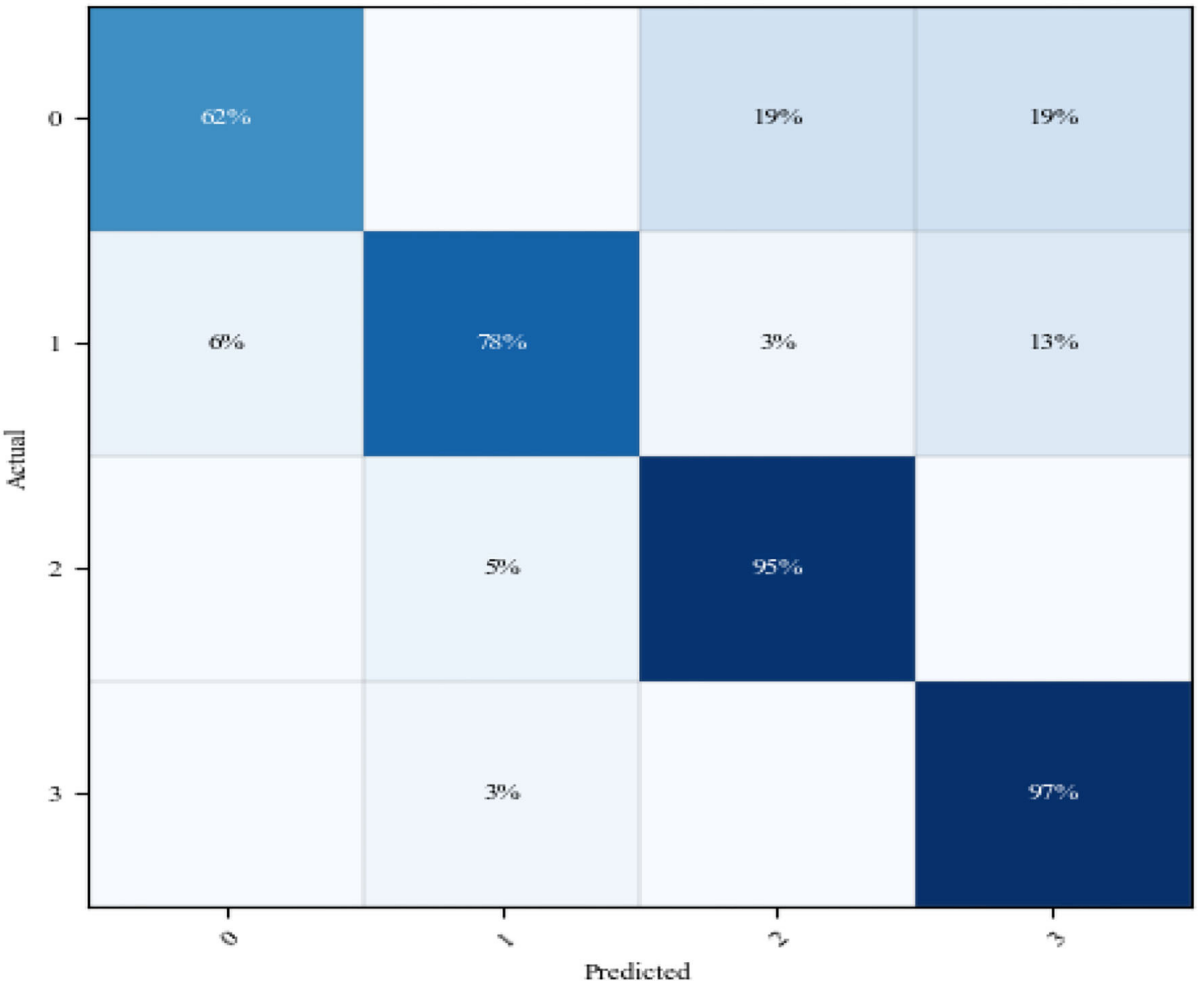
Confusion matrix for the modified-improved classification model during training with patients' dataset. Notes: in the two axes, 0 represents action 1, 1 represents action 2, 2 represents action 3, and 3 represents action 4.

Figure 10 shows that the accuracy of the classification model during the test with patient's dataset is 82.2%. Among the 107 extracted and preprocessed sEMG dataset, there are 26 action 1, 32 action 2, 19 action 3, and 30 action 4. Figure 11 shows the confusion matrix for the classification of this dataset.

From Figure 11, the classification accuracy of OT action 3 and OT action 4 is 95 and 97%, respectively. The prediction of OT action 2 is relatively moderate, with an accuracy rate of 78%. Some classes of OT action 2 are predicted and overlapped with OT action 4. The worse accuracy performance produced by the classification model is reported during the test of OT action 1, which is 62%. In this class, the main errors are concentrated in the error classification of OT actions 3 and 4. The poor classification performance for OT action 1 can be due to the reason that recruited patients have different severity levels. Also, execution of OT actions 1 and 3 during OT session is using similar muscle group in the upper limb. Due to variation in patients' severity level, some patients during the execution of OT action 3 may simulate action 1 instead. To improve the accuracy of this model over some OT's actions, large dataset for each stroke severity level should be collected. Further, the classification accuracy of the proposed upper limb classification system is comparable to that of an existing upper limb classification study of patients of the same size (14, 20).

### Performance Evaluation of Upper Limb Motion Completion

In the proposed upper limb motion completion, we fused IMUs and Kinect data with KF to effectively mitigate errors of IMU and overcome the instability of Kinect. The fusion results agreed with the finding in (22). Healthy participants' mobility data have been utilized as a benchmark to evaluate upper limb motion completion. Correlation between healthy subjects' mobility data shows a high CC. Thus, a motion data of a healthy individual when correlated with another healthy individual would be highly correlated. On the other hand, correlating the motion data of a patient with that of a healthy individual would have a lower CC based on the severity of the limb. Therefore, our hypothesis was that the correlation between the patient's motion data and that of a healthy subject would measure the quality and completion of the upper limb motion. In this study, kinematic data from healthy subjects and patients are correlated to estimate the joint's motion completion during four types of OT actions. The average Pearson CCs for each OT action are used as indictor to assess the OT action completion. Table 5 shows the average Pearson CCs for the nine patients during the execution of the four OT actions. The completion score for each OT action is calculated by mapping each CC to the corresponding range in Table 2.

**Table 5:** Average Pearson CCs for the nine patients during the four OT actions.

**Patient No**.	**Action 1**	**Action 2**	**Action 3**	**Action 4**
1	0.67	0.39	0.84	0.78
2	0.60	0.35	0.61	0.70
3	0.67	0.58	0.71	0.58
4	0.80	0.90	0.90	0.96
5	0.67	0.72	0.69	0.56
6	0.68	0.64	0.83	0.77
7	0.60	0.57	0.70	0.61
8	0.68	0.46	0.65	0.79
9	0.72	0.58	0.71	0.50

From Table 5, patients 1, 2, and 8 have CC values that are <0.5 when action 2 is executed, so their action completion score is 1 (mapped from Table 2). When executing the four OT actions, patients 3, 5, 6, 7, and 9 have CCs ranging from 0.5 to 0.8, so their action completion score is 2. The CCs of patient 4 for all four OT actions are greater than 0.80, so the action completion score for this patient is 3. The difference between CCs of other movements is modest, which is understandable as most patients have good performance in OT actions 1, 3, and 4. There is a high correlation between the CC results in this table and the muscle strength in Table 4. For example, using the Lovett muscle strength grading standard, patient 4 is rated as grade 5 which corresponds to his/her CC score of greater than 0.8. This patient performs similarly to a healthy subject. The proposed OT action completion method allows tracking of patient upper limb recovery progress like Brunnstrom's method for the same purpose (15, 16, 20, 27). The simplicity of the proposed method envisages improving communication efficiency with patients by allowing them to effortlessly understand their recovery progress.

**Figure 12:**
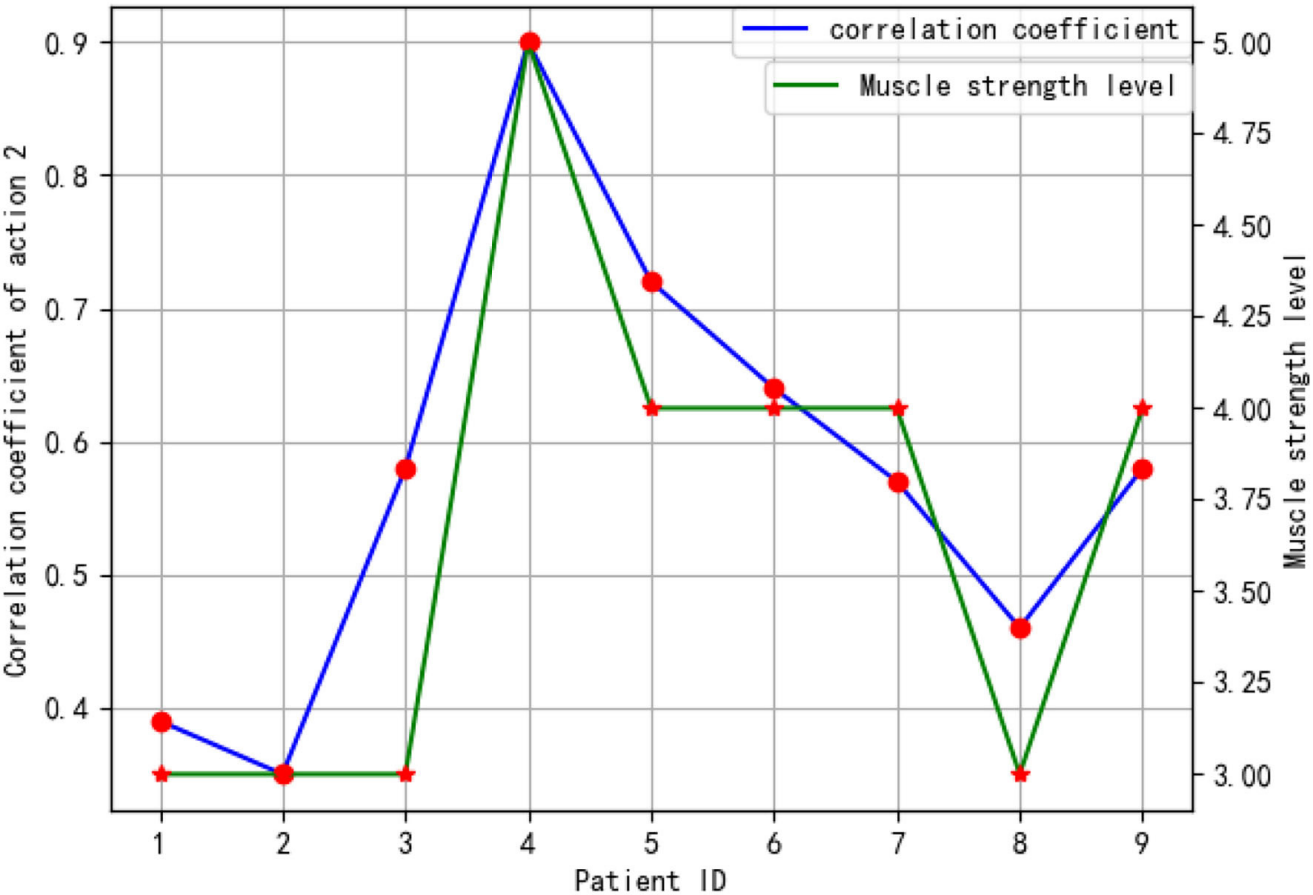
Correlation between patients' CC during OT action 2 and their muscle strength grade.

As a result of the correlation between the average coefficient of each OT action and patients' muscle strength grade, it was found that action 2 has a high correlation with muscle strength grade. Actions 1 and 3 have a moderate correlation, whereas action 4 has a low correlation. A low correlation between action 4 and muscle strength is caused by the difficulty of this action and the impairment severity of the patient. Through this action, the horizontal movement can be aided to some degree by the waist offset. It is a result of the frequent vertical movement in the air that can determine the movement capability of the upper limbs. Figure 12 shows patients' CCs during OT action 2 and their muscle strength grade. This figure represents the relation in the patients' CC and their muscle strength grade planes. The figure also shows that there is a high correlation between the patients' CC and their muscle strength grades.

Since the CCs for patients 3, 7, and 9 are similar, we used a DTW algorithm to calculate the distance between their movement dataset and the corresponding dataset from the healthy subjects. Table 6 shows the DTW distance results for each OT action. In OT action 2, based on the results in the table, the three patients produced distances that were similar. Furthermore, patient 3 outperformed the other two patients for all four OT actions.

**Table 6:** DTW measurement distance for the selected patients.

**Patient No**.	**Action 1**	**Action 2**	**Action 3**	**Action 4**
3	2.7	6.8	1.5	5.0
7	3.4	7.1	4.8	13.2
9	2.2	7.2	4.4	10.2

## Conclusion and Future Work

In this paper, a multisensor-based upper limb movement assessment method during OT has been proposed. Two kinematic sensors were fused to evaluate the completion of OT actions. The preprocessed sEMG and the modified improved TextCNN classifier have been used to classify the four OT actions.

The proposed upper limb OT actions' classification model has overall accuracy of 82.2 %. Kinect and Xsens fused data show a high correlation with muscle strength giving a CC of 0.88. Moreover, the experimental results show a high correlation between the patients' CC and their muscle strength grades.

The proposed upper limb movement assessment method can provide feedback to post-stroke patients about their upper limb motor improvement during the OT procedure, without a rehabilitation specialist intervention, thus improving the effectiveness of in-home OT. In the future, the improvement of the classification accuracy will be investigated. Large dataset for various upper extremities' severity levels is required to improve the accuracy.

## Data Availability Statement

The raw data supporting the conclusions of this article will be made available by the authors, without undue reservation.

## Ethics Statement

The experimental protocol was approved by the Ethics Committee of the Second Hospital of Jiaxing and was conducted in accordance with the Declaration of Helsinki. All participants signed a written informed consent before the experiment started. The patients/participants provided their written informed consent to participate in this study.

## Author Contributions

SM, ZC, and QF conceived and designed the experiments. ZC and JF performed the experiments. SM, ZC, JF, XG, and QF analyzed the data. All authors participated in writing the paper.

## Funding

This research was supported by a Li Ka-shing Foundation Cross-Disciplinary Research Grant (2020LKSFG01C).

## Conflict of Interest

The authors declare that the research was conducted in the absence of any commercial or financial relationships that could be construed as a potential conflict of interest.

## Publisher's Note

All claims expressed in this article are solely those of the authors and do not necessarily represent those of their affiliated organizations, or those of the publisher, the editors and the reviewers. Any product that may be evaluated in this article, or claim that may be made by its manufacturer, is not guaranteed or endorsed by the publisher.

## References

[1] N. S. Foundation. National Stroke Audit Rehabilitation Services Report 2012 Melbourne, VIC (2012).

[2] Bonita R Mendis S Truelsen T Bogousslavsky J Toole J Yatsu F. The global stroke initiative. Lancet Neurol. (2004) 3:391–3. 10.1016/S1474-4422(04)00800-2 15207791

[3] Report on Cardiovascular Health and Disease in China (2019) Excerpt 2: cerebral vascular disease. Prev Treat Cardiovasc Cerebrovasc Dis. (2020) 20:544–52. 10.3969/j.issn.1007-5410.2020.05.001

[4] Hankey GJ. Stroke. Lancet. (2017) 389:641–54. 10.1016/S0140-6736(16)30962-X 27637676

[5] Winstein CJ Stein J Arena R Bates B Cherney LR Cramer SC. Guidelines for adult stroke rehabilitation and recovery: a guideline for healthcare professionals from the American Heart Association/American Stroke Association. Stroke. (2016) 47:e98–169. 10.1161/STR.0000000000000098 27145936

[6] Wang G Wang T. Clinical Occupational Therapy Studies. Beijing, Huaxia Publishing House (2005).

[7] Guo N Chaomin N Zhang Y. Effect of occupational therapy on improving the daily living ability of stroke patients. Massage Rehabil Med. (2018) 9:10–1. 10.19787/j.issn.1008-1879.2018.16.005

[8] Qian H Huang Y Zhu S. Effects of early interventional occupational therapy on upper limb motor function and ADL ability in patients with acute stroke hemiplegia. Chin J Rhabil Med. (2007) 4:343–4. 10.3969/j.issn.1001-1242.2007.04.016

[9] Xu S Zhao J He L Shen M Cai X. Importance of occupational therapy in rehabilitation of stroke patients. Chin J Rehabil Theory Pract. (2012) 18:347–9. 10.3969/j.issn.1006-9771.2012.04.010

[10] Zhao S Cao Z. occupational therapy can improve self-efficacy and limb function of stroke patients. Genom Appl Biol. (2019) 38:933–9. 10.13417/j.gab.038.000933

[11] Richards LG Vallee C. Not Just Mortality and Morbidity but Also Function: Opportunities and Challenges for Occupational Therapy in the World Health Organization's Rehabilitation 2030. Can J Occup Ther. (2020) 87:91–9. 10.1177/0008417420910327 32180442

[12] Yanping R Qi G Yuqing L Jiahe Q Zhaoyang LV Bohan S. Current situation and demand of community rehabilitation medical resources in China. Chin J Rehabil Med. (2014) 29:757–9. 10.3969/j.issn.1001-1242.2014.08.015

[13] Fang Q Mahmoud S Gu X Fu J. A novel multi-standard compliant hand function assessment method using an infrared imaging device. IEEE J Biomed Health Inform. (2018) 23:758–65. 10.1109/JBHI.2018.2837380 29994552

[14] Lee S Lee YS Kim J. Automated evaluation of upper-limb motor function impairment using Fugl-Meyer assessment. IEEE Trans Neural Syst Rehabil Eng. (2018) 26:125–34. 10.1109/TNSRE.2017.2755667 28952944

[15] Zhang Z Fang Q Gu X. Objective assessment of upper-limb mobility for poststroke rehabilitation. IEEE Trans Biomed Eng. (2016) 63:859–68. 10.1109/TBME.2015.2477095 26357394

[16] Zhang Z Liparulo L Panella M Gu X Fang Q. A fuzzy kernel motion classifier for autonomous stroke rehabilitation. IEEE J Biomed Health Inform. (2016) 20:893–901. 10.1109/JBHI.2015.2430524 25956000

[17] Fang Q Mahmoud S Kumar A Gu X Fu J. A longitudinal investigation of the efficacy of supported in-home post-stroke rehabilitation. IEEE Access. (2020). 8:138690–700 10.1109/ACCESS.2020.301067427295638

[18] Ali A Thomas X Jafari R. Automatic noise estimation and context-enhanced data fusion of IMU and Kinect for human motion measurement. In: IEEE 14th International Conference on Wearable and Implantable Body Sensor Networks (BSN) Eindhoven: IEEE (2017). 10.1109/BSN.2017.7936036

[19] Wang C Peng L Hou Z-G Li J Zhang T Zhao J. Quantitative assessment of upper-limb motor function for post-stroke rehabilitation based on motor synergy analysis and multi-modality fusion. IEEE Trans Neural Syst Rehabil Eng. (2020) 28:943–52. 10.1109/TNSRE.2020.2978273 32149692

[20] Liparulo L Zhang Z Panella Gu X Fang Q. A novel fuzzy approach for automatic Brunnstrom stage classification using surface electromyography. Med Biol Eng Comput. (2017) 55:1367–78. 10.1007/s11517-016-1597-3 27909939

[21] Kim J Kim H Kim J. Quantitative assessment test for upper-limb motor function by using EMG and kinematic analysis in the practice of occupational therapy. Annu Int Conf IEEE Eng Med Biol Soc. (2017) 2017:1158–61. 10.1109/EMBC.2017.8037035 29060080

[22] Yushuang T Meng X Tao D. Dongquan L. Upper limb motion tracking with the integration of IMU and Kinect. Neurocomputing. (2015) 159:207–18. 10.1016/j.neucom.2015.01.071

[23] Kalman RE. A new approach to linear filtering and prediction problems. J Basic Eng. (1960), 82:35–45. 10.1115/1.366255230253628

[24] Vintsyuk TK. Speech discrimination by dynamic programming. Cybernetics. (1968) 4:52–7. 10.1007/BF01074755

[25] Kim Y. Convolutional neural networks for sentence classification. In: Proceedings of the 2014 Conf. on Empirical Methods in Natural Language Processing. Doha: Association for Computational Linguistics (2014). p. 1746–51. 10.3115/v1/D14-1181

[26] Hidayat A Arief Z Yuniarti H. LOVETT scalling with MYO armband for monitoring finger muscles therapy of post-stroke people. In: 2016 IEEE International Electronics Symposium (IES) Denpasar: IEEE (2016). 10.1109/ELECSYM.2016.7860977

[27] Shah S Harasymiw S Stahl P. Stroke rehabilitation: outcome based on Brunnstrom recovery stages. Occup Ther J Res. (1986) 6:365–76. 10.1177/153944928600600604

